# Case Report: Sublingual Microcirculatory Alterations in a Covid-19 Patient With Subcutaneous Emphysema, Venous Thrombosis, and Pneumomediastinum

**DOI:** 10.3389/fmed.2020.624695

**Published:** 2021-01-28

**Authors:** Sanjeev Grewal, Brita Harjo, Güclü Aykut, Bülent Ergin, Ralph Nowitzky, Can Ince, Sakir Akin

**Affiliations:** ^1^Department of Intensive Care, Haga Teaching Hospital, The Hague, Netherlands; ^2^Department of Intensive Care, Erasmus MC, University Medical Center Rotterdam, Rotterdam, Netherlands; ^3^Department of Cardiology, Erasmus MC, University Medical Center Rotterdam, Rotterdam, Netherlands

**Keywords:** COVID-19, pneumomediastinum, venous thrombosis, subcutaneous emphysema, sublingual microcirculation, IDF imaging

## Abstract

The Corona virus disease 2019 (Covid-19) has brought a wide range of challenges in intensive care medicine. Understanding of the pathophysiology of Covid-19 relies on interpreting of its impact on the vascular, particularly microcirculatory system. Herein we report on the first use of the latest generation hand-held vital microscope to evaluate the sublingual microcirculation in a Covid-19 patient with subcutaneous emphysema, venous thrombosis and pneumomediastinum. Remarkably, microcirculatory parameters of the patient were increased during the exacerbation period, which is not a usual finding in critically ill patients mostly presenting with a loss of hemodynamic coherence. In contrast, recovery from the disease led to a subsequent amelioration of these parameters. This report clearly shows the importance of microcirculatory monitoring for evaluating the course and the adequacy of therapy in Covid-19 patients.

## Introduction

The Corona virus disease 2019 (Covid-19) has brought a wide range of challenges in intensive care medicine. Understanding of the pathophysiology of Covid-19 primarily relies on interpreting of its effects on the vascular, particularly microcirculatory system (1).

In critically ill, microcirculatory alterations can be directly visualized at the bedside via handheld vital microscopy (HVM) (2). HVM devices, equipped with imaging techniques such as orthogonal polarization spectral (OPS) imaging and side-stream dark field (SDF) or incident dark field (IDF) imaging, have enabled real time microvascular monitoring in this cohort. Accordingly, microcirculatory alterations have been identified in advance of changes in systemic hemodynamic parameters in many clinical settings that are associated with cardiovascular compromise such as cardiac surgery and sepsis (3). Among HVM devices, Cytocam represents the newest generation IDF microscope which provides better image quality than the initially used devices (4).

Herein, we applied this technology to directly observe the sublingual microcirculation in a Covid-19 patient with subcutaneous emphysema, venous thrombosis and pneumomediastinum.

## Case Report

A 56-year-old man was admitted to our institution with an 11-day history of persistent dry cough, fever, and progressive shortness of breath. On arrival at the hospital, he was hypoxemic, with a SpO_2_ of 91% and a respiratory rate of 35 breaths per minute requiring ambient oxygen therapy with a flow of 15 L/min via a non-rebreather mask. Hemodynamically, he showed a stable profile with a blood pressure of 120/80 mmHg and a heart rate of 84 bpm. His body temperature was 38.1°C. Laboratory tests revealed an elevated C-reactive protein concentration of 37 mg/L (reference: <5 mg/L), a leukocyte count of 7.7 × 10^9^/L (reference: 4–10 × 10^9^/L) and D-dimer of 0.41 mg/L (reference: <0.50 mg/L). The chest radiograph demonstrated bilateral basal consolidations. The suspicion of Covid-19 was confirmed by real-time reverse transcription polymerase chain reaction (RT-PCR) analysis of nasopharyngeal swab samples.

**Figure 1:**
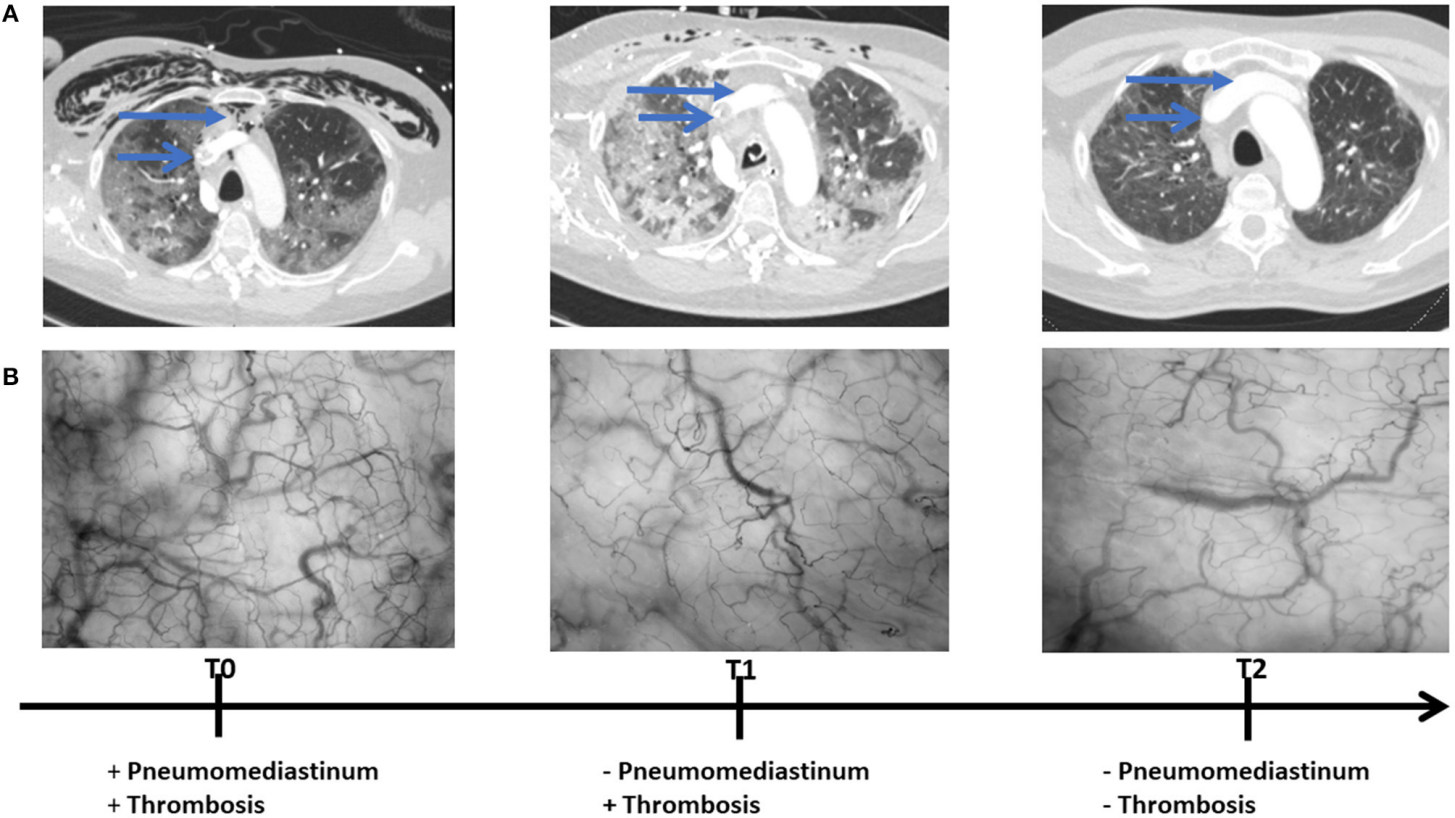
Chest CT **(A)** and microcirculatory images **(B)** obtained at 5th (T_0_), 19th (T_1_), and 25th (T_2_) day of intensive care admission.

The patient was primarily admitted to a designated Covid-19 ward for further observation and oxygen therapy. Due to severe hypoxemic respiratory failure within 24 h after admission, he was immediately transferred to the intensive care unit (ICU) where he was intubated. On the 5th day of ICU admission (T_0_), the patient developed a high fever accompanied with an increase in inflammatory parameters (Table 1). Hemodynamic parameters did not indicate an unstable course (Table 1). Microcirculatory monitoring was carried out sublingually using Cytocam-IDF imaging (CytoCam, Braedius Medical, Huizen, The Netherlands). Image analysis which was performed offline using MicroTools, showed an increase in microcirculatory density (total vessel density and perfused vessel density) and perfusion parameters (proportion of perfused vessels and microvascular mean flow index) as well as in red blood cell (RBC) velocity (Table 2). Subsequent CT-scan demonstrated extensive subcutaneous emphysema and pneumomediastinum as well as a thrombus in the right jugular vein extending to the superior vena cava and into the right brachial vein (Figure 1). A tracheal defect was ruled out by bronchoscopy. There was no pneumothorax or evidence of esophageal rupture.

**Table 1:** Case timeline.

**Variable**	**T_0_**	**T_1_**	**T_2_**
Day of onset of Covid-19 symptoms	16	30	36
Day of intensive care unit admission	5	19	25
Heart rate, beats/min	81	129	106
Mean arterial pressure, mmHg	105/56	126/61	136/79
PaO_2_/FiO_2_, mmHg	115.5	112.5	174.3
Mode of ventilation	PCV	PCV	PSV
Tidal volume, ml	450	348	446
Positive end-expiratory pressure, cmH_2_O	16	6	5
Fraction of inspired oxygen, %	50	50	40
Driving pressure, cmH_2_O	14	24	9
Compliance respiratory system, ml/cmH_2_O	18.8	14.5	27
Temperature, °C	37.5	37.2	38.3
Fluid balance, ml	270	−2,000	−2,600
Creatinine, μmol/L	75	96	48
C-reactive protein, mg/L	213	226	382
Hematocrit, %	41	30	34
Thrombocyte count, ×10^9^/L	473	483	144
Leukocyte count, ×10^9^/L	14.2	17.8	16.3
pH	7.46	7.32	7.36
PaCO_2_, kPa	5.8	7.7	8.4
PaO_2_, kPa	7.7	7.5	9.3
Bicarbonate, mmol/L	30	27	31
Base excess, mmol/L	6.7	3.7	8.8
Oxygen saturation, %	90	90	94
Lactate, mmol/L	1.9	0.6	0.8

*T, time point; COVID-19, Coronavirus Disease 2019; PaCO_2_, partial pressure of carbon dioxide; PaO_2_, partial pressure of oxygen; PCV, pressure-controlled ventilation; PSV, pressure-supported ventilation*.

**Table 2:** Microcirculatory parameters.

**Variable**	**T_0_**	**T_1_**	**T_2_**
FCD, mm/mm^2^	29.61 ± 0.92	28.02 ± 3.0	21.74 ± 3.84
PPV, %	95 ± 2	96 ± 2	98 ± 1
RBCv, μm/s	298.13 ± 19.61	327.10 ±18.89	354.81 ± 23.89
TVD, mm/mm^2^	31.24 ± 1.24	29.23 ± 2.67	22.15 ± 3.82

*Values are presented as mean ± standard deviation*.

*T, time point; FCD, functional capillary density; PPV, proportion of perfused vessels; RBCv, red blood cell velocity; TVD, total vessel density*.

The patient was ventilated for 19 days and a temporary tracheostomy was performed for a gradual wean from the ventilator. Unfractionated heparin was given in therapeutic doses for his venous thrombus. Chest CT obtained at 19th (T_1_) and 25th day of ICU admission (T_2_) showed complete resorption of mediastinal air and venous thrombus (Figure 1). Concurrent microcirculatory images revealed a recovery of microcirculatory alterations (Table 2 and Figure 1). Thereupon on the 30th day of ICU admission, the tracheostomy was removed and the patient was discharged to the ward after 32 days of ICU stay in stable hemodynamic and respiratory condition.

## Discussion

To our knowledge this is the first case report demonstrating microcirculatory alterations in a Covid-19 patient with subcutaneous emphysema and pneumomediastinum. Remarkably, microcirculatory parameters and RBC velocity of the patient showed an increase during the exacerbation period, which is not a usual finding in critically ill patients mostly presenting with a loss of hemodynamic coherence (3). In contrast, recovery from the disease led to a subsequent amelioration of these parameters.

The increase in microcirculatory parameters and RBC velocity in this patient may be explained by an intact compensatory mechanism of the microcirculation capable of responding to hypoxia (5). This compensatory mechanism is aimed at increasing the oxygen extraction capacity of the microcirculation by decreasing diffusion distances between capillaries (increased TVD) and by increasing convection of RBCs (increased RBC flow). Similar microcirculatory response to hypoxia has been reported in recent microcirculation studies which demonstrated increased sublingual microcirculatory vessel density as a response to hypoxic conditions at high altitude (6, 7). Notably, this intact compensatory mechanism may also explain the ability of Covid-19 patients to cope with low levels of oxygen which has been described as “happy hypoxia” (8).

Indeed, contrary to our results, Edul et al. (9) showed a decline in RBC velocity accompanied by an increase in vessel density in patients suffering from Covid-19 pneumonia. Furthermore, Rovas et al. (10) noted reductions in RBC velocity with decreases in vessel density in this cohort. Since hypoxemia induces both capillary recruitment and angiogenesis (9), differences might be related to the higher compromise of pulmonary oxygenation in our patient [PaO_2_/FiO_2_ ratio, 112.5 mmHg vs. 122 ± 43 mmHg (9) vs. 194.88 (145.76–234.0) mmHg (10)]. Spontaneous pneumomediastinum associated with Covid-19 has occasionally been reported (11). The presumed etiology involves a virus induced diffuse alveolar damage during an increase in intra-alveolar pressure, leading to alveolar rupture, a mechanism known as the Macklin effect (12). Ultimately, this pathology together with venous thrombus might have also influenced microcirculatory parameters in our patient, amplifying the microcirculatory response to hypoxia. Furthermore, the increase in microcirculatory parameters might have only been caused by these pathologies.

The main limitation of our study concerns the small sample size, thus making it difficult to generalize conclusions. Although in our patient a spontaneous pneumomediastinum was associated with a severe course of COVID-19 pneumonia, it is yet unclear to what extend the increase in microcirculatory parameters might predict the severity of disease in Covid-19 patients who don't present with pneumomediastinum. Notwithstanding the limitations of our study, we obtained a valuable patient perspective. Overall, our patient felt well and did not have any complications. Remarkably, he appreciated the care he received in our hospital.

In conclusion, this report clearly shows the importance of microcirculatory monitoring for evaluating the course and the adequacy of therapy in Covid-19 patients. Future studies are warranted to assess whether microcirculatory parameters could find potential clinical use as a predictor of the severity of disease in this cohort.

## Data Availability Statement

The original contributions presented in the study are included in the article/Supplementary Materials, further inquiries can be directed to the corresponding author/s.

## Ethics Statement

Ethical approval was not required according to the local legislation and institutional guidelines. Written informed consent was obtained from the patient for the publication of any potentially identifiable images or data included in this article.

## Author Contributions

SG, BH and RN performed the IDF imaging and contributed to the manuscript revision. GA participated in the interpretation of the data, and drafted and revised the manuscript. BE was involved in the image analysis and the manuscript revision. CI and SA participated in the design of the study and contributed to the manuscript revision. All authors read and approved the final version of the manuscript.

## Conflict of Interest

CI has developed SDF imaging and is listed as an inventor on related patents that were commercialized by Micro Vision Medical (MVM) under a license from the Academic Medical Center (AMC). He receives no royalties or benefits from this license. He has been a consultant for MVM in the past but has not been involved with this company for more than five years and holds no shares or stock. Braedius Medical, which is a company that is owned by a relative of CI, has developed and designed a handheld microscope, namely, the CytoCam-IDF imaging microscope. The images used in the present study were obtained using this technology. CI has no financial relationship with Braedius Medical of any sort. He has never owned shares or received consultancy or speaker fees from this company. MicroTools software that was used for analysis of the images is being developed by CI and Dr. Mathias Hilty and owned by Active Medical BV Leiden, The Netherlands. Active Medical also runs an internet site called microcirculationacademy.org which offers educational courses and services that are related to clinical microcirculation. CI discloses that he is a shareholder of this company. The remaining authors declare that they do not have any commercial or financial relationships that could be construed as a potential conflict of interest.
